# Cost-effectiveness of ace inhibitors versus ARBs in heart failure management

**DOI:** 10.1097/MD.0000000000039496

**Published:** 2024-09-06

**Authors:** Chukwuka Elendu, Dependable C. Amaechi, Tochi C. Elendu, Emmanuel C. Amaechi, Ijeoma D. Elendu, Klein A. Jingwa, Sobechukwu F. Chiegboka, Umesh Bhadana, Abdelrahman M.S. Abdelatti, Ifeanyi V. Ikeji, Jadzia C. Atmadibrata, Ahmed S.F. Mohamed, Umar Janibabu Sharmila, Fathy E.A.E. Soltan, Nada K. Abbas, Mariam M.F. Eldorghamy, Tuvakbibi Gurbanova, Arome K.B. Okeme, Arinze A. Okeke, Ikpembhosa J. Esangbedo

**Affiliations:** a Federal University Teaching Hospital, Owerri, Nigeria; b Igbinedion University, Okada, Nigeria; c Imo State University, Owerri, Nigeria; d Madonna University, Elele, Nigeria; e Kazan State Medical University, Kazan, Russia; f Vinnytsia National Medical University, Vinnytsia, Ukraine; g Ternopil State Medical University, Ternopil, Ukraine; h Bayero University, Kano, Nigeria; i Federal Teaching Hospital, Ido-ekiti, Nigeria; j University College Hospital, Ibadan, Nigeria.

**Keywords:** ACE inhibitors, ARBs, cost-effectiveness, heart failure, incremental cost, QALY (quality-adjusted life year), sensitivity analysis

## Abstract

**Background:**

Heart failure is a chronic condition that imposes a significant burden on healthcare systems worldwide. Effective management is crucial for improving patient outcomes and reducing costs. Angiotensin-converting enzyme (ACE) inhibitors and angiotensin II receptor blockers (ARBs) are widely used to manage heart failure by reducing cardiac strain and preventing disease progression. Despite their common use, ACE inhibitors and ARBs differ in mechanisms, cost, and potential side effects. ACE inhibitors have long been the standard treatment, while ARBs are often prescribed to patients intolerant to ACE inhibitors, particularly due to side effects like cough. Given these differences, evaluating the cost-effectiveness of these treatments is essential. This study compares the cost-effectiveness of ACE inhibitors and ARBs from a healthcare system perspective, considering both direct medical costs and health outcomes.

**Methods:**

A cost-effectiveness analysis was conducted using a decision-analytic Markov model to simulate heart failure progression in a hypothetical cohort. Data inputs included clinical trial outcomes, real-world effectiveness data, direct medical costs (medications, hospitalizations, monitoring), and utility values for quality of life. The primary outcome measures were the cost per quality-adjusted life year gained and the incremental cost-effectiveness ratio. Sensitivity analyses tested the robustness of results, and subgroup analyses were conducted based on age and disease severity.

**Results:**

The base-case analysis showed that ACE inhibitors were associated with lower overall costs and slightly higher quality-adjusted life years than ARBs. Sensitivity analyses revealed that variations in key parameters, such as transition probabilities, mortality rates, and healthcare expenses, had limited impact on the overall cost-effectiveness conclusions. Subgroup analyses indicated that ACE inhibitors and ARBs exhibited similar cost-effectiveness profiles for patients aged <65 and ≥65 years. However, among patients with severe heart failure, ARBs demonstrated a higher incremental cost-effectiveness ratio compared with ACE inhibitors, suggesting reduced cost-effectiveness in this subgroup.

**Conclusion:**

ACE inhibitors are likely a more cost-effective option for managing heart failure than ARBs, particularly from a healthcare system perspective. The findings underscore the importance of tailoring treatment decisions to individual patient factors, preferences, and clinical conditions, providing valuable insights for healthcare policy and practice, particularly regarding cost-effectiveness across patient subgroups.

## 1. Introduction

Heart failure is a prevalent and serious condition that presents significant health and economic challenges worldwide. It is a major cause of morbidity and mortality, and its management requires effective and evidence-based treatment strategies.^[1,2]^ Among the pharmacological options, angiotensin-converting enzyme (ACE) inhibitors and angiotensin receptor blockers (ARBs) are commonly prescribed for heart failure patients to reduce symptoms, improve quality of life, and decrease mortality. Both classes of drugs work by inhibiting the renin-angiotensin-aldosterone system, but they do so via different mechanisms.^[3,4]^ The choice between ACE inhibitors and ARBs in clinical practice can be influenced by several factors, including efficacy, safety profile, patient tolerance, and cost. ACE inhibitors are generally considered first-line therapy, but some patients may not tolerate them due to adverse effects like cough or angioedema. ARBs, which block the angiotensin II receptor, are often used as an alternative in such cases.^[5,6]^ Given the differences in mechanism, side effect profiles, and potentially differing costs, it is crucial to determine which class of drug offers the best value for money in the management of heart failure. The comparative cost-effectiveness of ACE inhibitors and ARBs has important implications for clinical decision-making, healthcare resource allocation, and patient outcomes.^[7,8]^ Thus, comparing these 2 strategies can provide valuable insights into optimizing treatment protocols and ensuring that patients receive the most effective and efficient care available.^[9–11]^

### 1.1. Background

Heart failure, characterized by the heart’s inability to pump blood effectively, manifests as a dynamic interplay of states rather than a static condition.^[3]^ Continuous progression, interspersed with periods of stability and unpredictable exacerbations requiring hospitalization, defines the intricate landscape of heart failure. Unlike the rigid transitions in static models, heart failure follows a fluid trajectory influenced by various factors, challenging conventional analytical approaches.^[4]^

### 1.2. Objectives

This paper aims to scrutinize the limitations of traditional static models in capturing the nuances of heart failure’s dynamic nature. By exploring the continuous evolution of heart failure states, we seek to address the inadequacies of conventional models and pave the way for a more comprehensive understanding of this intricate cardiovascular condition.^[5]^

### 1.3. Study question

How well do traditional static models align with the dynamic and unpredictable course of heart failure? This study endeavors to answer this crucial question, emphasizing the need for analytical approaches that mirror the fluidity inherent in heart failure progression.^[6]^

### 1.4. Relevance for health policy and practice decisions

Recognizing the dynamic nature of heart failure is not merely an academic pursuit; it holds profound implications for health policies and clinical practices.^[7]^ Inaccuracies in modeling can lead to suboptimal resource allocation and misguided treatment strategies. Acknowledging the dynamic aspects is imperative for informed decision-making, ensuring that healthcare policies resonate with the ever-changing landscape of heart failure.^[8]^

## 2. Methods

### 2.1. Target population

The base case population for this study comprises adult patients diagnosed with heart failure. These patients are assumed to have a range of heart failure severity, from mild to severe, and are representative of the broader heart failure population typically encountered in clinical practice.

### 2.2. Subgroups analyzed

1. **Severity subgroups:** To account for variations in heart failure severity, the study includes subgroups representing patients with mild, moderate, and severe heart failure. These subgroups are chosen to capture the diverse spectrum of patients seen in clinical practice. Patients are classified based on established clinical criteria, including left ventricular ejection fraction (LVEF) and symptom severity.
2. **Age subgroups:** Age is an essential factor in heart failure management. The study analyzes subgroups based on age categories, such as young adults (18–45 years), middle-aged adults (46–65 years), and older adults (65+ years). This stratification recognizes that treatment decisions and outcomes may differ across age groups.
3. **Comorbidity subgroups:** Heart failure often coexists with other comorbid conditions, such as hypertension or diabetes. Subgroups are analyzed based on specific comorbidities to assess how these conditions may influence treatment outcomes and cost-effectiveness.
4. **Medication adherence subgroups:** Medication adherence is critical to heart failure management. Subgroups are formed based on varying levels of medication adherence, ranging from high to low. This analysis aims to understand the impact of adherence on cost-effectiveness.
5. **Gender subgroups:** Gender-based differences in heart failure incidence and response to treatment have been observed. Therefore, the study includes gender subgroups (male and female) to explore potential disparities in cost-effectiveness.

These subgroups are selected based on clinical relevance and the need to account for the heterogeneity within the heart failure population. Analyzing these subgroups allows for a more comprehensive evaluation of the cost-effectiveness of ACE inhibitors versus ARBs in various clinical scenarios and patient profiles. This approach helps provide tailored insights for clinicians and policymakers who must make decisions considering the diverse patient populations encountered in real-world practice.

### 2.3. Setting and location

The decision-making context for this study is situated within a typical healthcare system, which encompasses the following relevant aspects:

1. **Healthcare infrastructure:** The study considers the availability and accessibility of healthcare facilities, including hospitals, clinics, and primary care providers. The infrastructure’s capacity to manage heart failure and related services is integral to decision-making.
2. **Healthcare providers:** Decisions regarding heart failure management involve various healthcare providers, including cardiologists, internists, nurses, and pharmacists. The study acknowledges the roles and perspectives of these providers in the decision-making process.
3. **Insurance and reimbursement systems:** The healthcare system’s insurance and reimbursement mechanisms play a crucial role in determining the financial implications of treatment choices. This includes public and private insurance programs and reimbursement policies for medications.
4. **Regulatory framework:** The study operates within the regulatory framework governing the approval, prescription, and usage of medications like ACE inhibitors and ARBs. Compliance with regulatory guidelines and policies is essential in decision-making.
5. **Patient access and preferences:** Patient factors, such as access to healthcare services, socioeconomic status, and individual preferences, influence treatment decisions. The study accounts for these aspects, recognizing that patient-centered care is integral to healthcare decision-making.
6. **Health policy landscape:** Healthcare policies and guidelines set by government health agencies and professional medical organizations provide a framework for clinical practice and reimbursement. The study considers these policies when evaluating the cost-effectiveness of treatment options.
7. **Geographic location:** Geographic factors, including urban and rural disparities, healthcare resource distribution, and regional healthcare delivery models, may impact decision-making. The study acknowledges these variations in healthcare delivery based on location.
8. **Economic factors:** Economic conditions, including healthcare budgets, cost containment strategies, and resource allocation, influence the affordability and sustainability of heart failure management strategies.

### 2.4. Study perspective

The study adopts a healthcare system or payer perspective. This means that the analysis primarily focuses on costs and outcomes that are relevant and directly incurred by the healthcare system or payer responsible for covering the expenses associated with heart failure management. The critical elements of this perspective include:

1. **Direct medical costs:** The analysis considers direct medical costs, such as medications (ACE inhibitors and ARBs), hospitalizations, outpatient visits, diagnostic tests, and monitoring. These costs are incurred by the healthcare system when providing care to heart failure patients.
2. **Health outcomes:** The study evaluates health outcomes regarding quality-adjusted life years (QALYs) gained. QALYs consider both the quantity and quality of life experienced by patients, allowing for a comprehensive assessment of treatment effectiveness.
3. **Costs of medications:** The costs associated with the 2 treatment options, ACE inhibitors and ARBs, are explicitly considered. This includes the cost of acquiring and administering these medications to patients within the healthcare system.
4. **Costs of hospitalizations and healthcare services:** The study assesses the costs of hospital admissions, emergency room visits, and other healthcare services directly related to heart failure management. The healthcare system bears these costs.
5. **Monitoring and follow-up costs:** Costs associated with patient monitoring, follow-up appointments, and any additional interventions are included. These costs are part of the overall expenses incurred by the healthcare system.

#### 2.4.1. Relation to costs being evaluated

The healthcare system or payer perspective explicitly evaluates costs relevant to the entities responsible for financing and delivering healthcare services. By adopting this perspective, the analysis ensures that decision-makers, such as healthcare policymakers and payers, clearly understand the financial implications of choosing ACE inhibitors or ARBs for heart failure management. This information is vital for optimizing resource allocation, budget planning, and making informed decisions about which treatment strategy provides the best value in cost-effectiveness.

### 2.5. Interventions/comparators

1. **ACE inhibitors (angiotensin-converting enzyme inhibitors):**
  • ACE inhibitors are a class of medications that block the action of angiotensin-converting enzyme, which plays a role in constricting blood vessels and retaining salt and water. ACE inhibitors help relax blood vessels, reduce fluid buildup, and lower blood pressure by inhibiting this enzyme.
  • ACE inhibitors, such as enalapril and lisinopril, have been widely used in heart failure management and have a substantial body of clinical evidence supporting their efficacy.
2. **ARBs (angiotensin receptor blockers):**
  • ARBs, also known as angiotensin receptor antagonists, block the action of angiotensin II, a hormone that narrows blood vessels and increases blood pressure. ARBs help relax blood vessels and reduce the strain on the heart.
  • Common ARBs include losartan and valsartan. These medications are also commonly prescribed for heart failure.

### 2.6. Reasons for their selection

1. **Clinical relevance:** ACE inhibitors and ARBs are established and recommended heart failure treatments. They target the renin-angiotensin-aldosterone system, which is pivotal in heart failure pathophysiology. Comparing these 2 classes of medications is clinically relevant because they are often considered first-line therapies.
2. **Common clinical practice:** ACE inhibitors and ARBs are commonly used in real-world clinical practice for heart failure management. This analysis reflects the choices clinicians and healthcare providers routinely face when determining treatment plans for heart failure patients.
3. **Comparable efficacy:** ACE inhibitors and ARBs have shown efficacy in improving heart failure symptoms, reducing hospitalizations, and enhancing patients’ quality of life. However, there may be differences in their cost-effectiveness profiles, making it essential to compare them systematically.
4. **Resource allocation:** Given the limited resources within healthcare systems, assessing which of these 2 interventions provides better value for the investment is crucial. Understanding their cost-effectiveness can aid in resource allocation decisions.

### 2.7. Time horizon

The study employs a long-term time horizon, typically several years, to evaluate costs and consequences. A typical time horizon might be 5 to 10 years, although it can vary depending on specific study objectives and healthcare contexts.

#### 2.7.1. Reasons for a long-term time horizon

1. **Chronic nature of heart failure:** Heart failure is a chronic condition with ongoing management and potential changes in the patient’s health status over time. A long-term time horizon allows for assessing treatment strategies’ effectiveness and cost implications throughout the natural course of the disease.
2. **Delayed clinical outcomes:** The impact of ACE inhibitors and ARBs on heart failure outcomes, such as hospitalizations, mortality, and quality of life, may take time to manifest fully. A long-term perspective ensures that the study captures these delayed consequences.
3. **Resource allocation:** Healthcare decision-makers need information on the long-term cost implications of treatment choices to make informed decisions about resource allocation within healthcare systems. This includes budget planning and assessing the sustainability of interventions over time.
4. **Clinical guidelines and recommendations:** Clinical guidelines often consider long-term outcomes and recommend effective treatment strategies over extended periods. The study’s time horizon aligns with the clinical guideline recommendations for heart failure management.
5. **Policy and budgetary planning:** Healthcare policymakers and payers require data on the long-term cost-effectiveness of interventions to inform policy decisions and allocate healthcare budgets effectively.

### 2.8. Discount rates

1. **Costs:** A discount rate is applied to future costs to bring them to their present value. The standard discount rate for costs in healthcare economic evaluations is often in the range of 3% to 5%, depending on the healthcare system and guidelines in the specific region where the study is conducted.
2. **Outcomes (health benefits):** Similarly, a discount rate is applied to future health outcomes (e.g., quality-adjusted life years, QALYs) to reflect their present value. The same discount rate for costs is typically used to maintain consistency and ensure comparability.

#### 2.8.1. Reasons for the choice of discount rates:

1. **Time preference:** Discounting accounts for people’s time preference for benefits received today over benefits received in the future. In healthcare, it acknowledges that resources spent today have an opportunity cost and could be invested elsewhere.
2. **Consistency with guidelines:** Many healthcare economic guidelines and recommendations stipulate using a specific discount rate, often in the abovementioned range, to maintain consistency in economic evaluations and facilitate decision-making.
3. **Policy and budgetary planning:** The chosen discount rates align with the typical rates used by healthcare policymakers and payers when assessing the cost-effectiveness of interventions. This ensures that the study’s findings are relevant for resource allocation and policy decisions.
4. **Transparent and standardized analysis:** Using standard discount rates enhances the transparency and comparability of the analysis. It allows stakeholders to assess the study’s methodology and results more quickly.

### 2.9. Outcome measure

1. **Quality-adjusted life year (QALY):**
  • A QALY is a widely accepted measure in health economics that combines quantity and quality of life into a single metric.
  • It quantifies the impact of an intervention on a patient’s overall well-being by accounting for changes in health-related quality of life and life expectancy.
  • QALYs are typically measured on a scale from 0 to 1, where 0 represents a health state equivalent to death, and 1 illustrates perfect health. Health states between 0 and 1 reflect various degrees of impaired health-related quality of life.
  • The change in QALYs before and after treatment provides a comprehensive assessment of the intervention’s impact on the patient’s health and allows for comparisons of different treatments.

#### 2.9.1. Relevance for the analysis

The choice of QALYs as the measure of benefit in this analysis is highly relevant for the following reasons:

1. **Comprehensive health assessment:** Heart failure is a condition that affects both the quantity and quality of life. QALYs capture the full spectrum of health outcomes, from symptoms and functional status changes to the potential life extension.
2. **Comparison of interventions:** QALYs allow for direct comparisons between ACE inhibitors and ARBs regarding their impact on patients’ overall well-being. This facilitates assessing which treatment strategy provides a more significant benefit than cost.
3. **Patient-centered approach:** QALYs incorporate patients’ preferences and values regarding their health. This patient-centered approach aligns to optimize patient outcomes and satisfaction.
4. **Alignment with health economic guidelines:** QALYs are recommended by health economic guidelines and are commonly used in cost-effectiveness analyses. Their widespread use ensures that the results of this analysis can be compared and integrated into healthcare decision-making processes.

### 2.10. Single study-based estimates

In the context of this cost-effectiveness analysis comparing ACE inhibitors to ARBs in heart failure management, let’s consider the design features of a hypothetical single effectiveness study and why it might be considered a sufficient source of clinical effectiveness data:

#### 2.10.1. Design features of the single effectiveness study

- **Study design:** The single effectiveness study is a randomized controlled trial (RCT), considered the gold standard for evaluating treatment efficacy.
- **Patient population:** The study includes a representative sample of heart failure patients from different age groups and severity levels (mild, moderate, severe). Patients have been carefully diagnosed and stratified.
- **Interventions:** The study compares the effectiveness of ACE inhibitors to ARBs in heart failure management. Patients are randomly assigned to one of these treatment groups.
- **Outcome measures:** The study collects comprehensive data on relevant clinical outcomes, including mortality rates, hospitalization rates, symptom improvement, and quality of life measures assessed through validated scales.
- **Follow-up duration:** The study follows patients over a sufficiently long duration, typically several years, to capture both short-term and long-term treatment effects.

#### 2.10.2. Why the single study is sufficient

The single RCT described above may be considered a sufficient source of clinical effectiveness data for several reasons:

1. **Internal validity:** RCTs are known for their rigorous design, which minimizes bias and confounding factors, ensuring high internal validity.
2. **Direct comparison:** The study directly compares the 2 interventions of interest (ACE inhibitors and ARBs) in a controlled setting, providing head-to-head effectiveness data.
3. **Diverse patient population:** Including patients with varying degrees of heart failure severity and across different age groups enhances the study’s generalizability.
4. **Comprehensive outcome measures:** The study collects a broad range of clinical outcomes relevant to heart failure management, allowing for a thorough assessment of treatment effectiveness.
5. **Longitudinal data:** The study’s extended follow-up duration captures short-term and long-term treatment effects, aligning with the analysis’s long-term time horizon.
6. **Relevance to clinical practice:** Findings from a well-designed RCT are highly relevant to clinical practice and decision-making, making it a valuable source of clinical effectiveness data.

### 2.11. Synthesis-based estimates

If multiple studies are included to synthesize clinical effectiveness data, the methods used for the identification and synthesis of these studies would typically involve the following steps:

#### 2.11.1. Identification of included studies

1. **Literature search:** A systematic and comprehensive literature search uses relevant databases (e.g., PubMed, Cochrane Library) to identify all relevant studies comparing ACE inhibitors and ARBs in heart failure management.
2. **Inclusion criteria:** Studies meeting predetermined inclusion criteria are selected. These criteria may include study design (e.g., RCTs), patient population (e.g., heart failure patients), interventions (ACE inhibitors and ARBs), and outcome measures.
3. **Data extraction:** Relevant data, including study characteristics, patient demographics, intervention details, and outcome measures, are extracted from each selected study.

#### 2.11.2. Synthesis of clinical effectiveness data

1. **Quantitative analysis:** If data from multiple studies with similar outcome measures are available, a meta-analysis may be conducted to pool the results statistically. This allows for estimating summary effect sizes (e.g., risk ratios, mean differences) and their associated confidence intervals.
2. **Heterogeneity assessment:** The heterogeneity of included studies is assessed to determine the degree of variation in results. Subgroup analyses or sensitivity analyses may be performed to explore potential sources of heterogeneity.
3. **Publication bias:** Publication bias occurs when studies with significant results are more likely to be published and are assessed using statistical tests or visual inspection of funnel plots.
4. **Quality assessment:** The quality and risk of bias of included studies are evaluated to ascertain the overall strength of the evidence.

#### 2.11.3. Population

The population for eliciting preferences for outcomes would typically include heart failure patients or individuals representing the target population. The selection of participants would aim for diversity in age, the severity of heart failure, and other relevant characteristics to capture a representative range of preferences.

#### 2.11.4. Methods for eliciting preferences

Preference-based outcomes are commonly measured using the following methods:

1. **EuroQol 5-Dimension (EQ-5D) questionnaire:** The EQ-5D is a widely used instrument for assessing HRQoL and eliciting preferences for health states. It comprises 5 dimensions (mobility, self-care, usual activities, pain/discomfort, anxiety/depression), and participants rate their health on each dimension. The resulting health states are assigned utility values, typically on a scale from 0 (representing death) to 1 (representing perfect health).
2. **Time trade-off (TTO):** TTO is a direct preference elicitation method where individuals are asked to trade off time in their current health state for time in perfect health. It provides a numerical value reflecting an individual’s preference for their everyday health state.
3. **Standard gamble (S.G.):** S.G. is another direct preference elicitation method where individuals are presented with a choice between their current health state and a risky gamble involving a chance of total health or death. The utility value is derived based on the individual’s willingness to take the risk.
4. **Visual analog scale (VAS):** In VAS, individuals rate their overall health on a scale typically ranging from 0 (worst imaginable health) to 100 (best imaginable health). This provides a single-point measure of their health-related quality of life.

#### 2.11.5. Relevance for the analysis

Preference-based outcomes, such as utility values derived from the EQ-5D or direct preference elicitation methods, are highly relevant for cost-effectiveness analysis. They allow for the calculation of Quality-Adjusted Life Years (QALYs), which combine changes in the length and quality of life associated with different treatment strategies. QALYs are a standard measure in cost-effectiveness analyses, enabling comparisons across various healthcare interventions and conditions.

#### 2.11.6. Single study-based economic evaluation

In a single study-based economic evaluation comparing ACE inhibitors to ARBs in heart failure management, the following approaches are used to estimate resource use associated with the alternative interventions:

#### 2.11.7. Approaches to estimate resource use

1. **Clinical data:** Resource use data are typically collected alongside clinical data within the same study. This involves tracking various healthcare resource utilization by patients, such as hospitalizations, outpatient visits, diagnostic tests, and medication usage.
2. **Patient records:** Medical records and billing data may be used to extract information on resource use. These records can provide detailed information on the types and frequencies of healthcare services received by patients.

#### 2.11.8. Valuing resource items in terms of unit cost

For each resource item, primary or secondary research methods are used to assign unit costs:

1. **Primary data collection:** In some cases, the study may collect resource cost data directly. For instance, the cost of medications (ACE inhibitors and ARBs) can be obtained from pharmacy records or pricing databases.
2. **Secondary data sources:** Unit costs for resource items can also be sourced from established databases, such as healthcare reimbursement databases, government healthcare cost databases, or published literature. These sources provide standardized cost information.
3. **Healthcare providers:** Costs of healthcare services provided by hospitals, clinics, and healthcare professionals can be obtained by contacting healthcare providers directly or through reimbursement data.

#### 2.11.9. Adjustments to approximate opportunity costs

To approximate opportunity costs, adjustments may be made to the unit costs:

1. **Discounting:** Costs incurred in the future are typically discounted to their present value using a discount rate (as discussed earlier) to account for the time value of money.
2. **Inflation:** If necessary, unit costs may be adjusted for inflation to express them in constant currency terms, ensuring that prices are comparable over time.
3. **Market prices:** Unit costs are often based on market prices or reimbursement rates, which approximate the opportunity cost of resources in the healthcare system.

#### 2.11.10. Model-based economic evaluation

In a model-based economic evaluation, where a decision-analytic model is used to estimate resource use associated with model health states, the following approaches and data sources are typically used:

#### 2.11.11. Approaches to estimate resource use

1. **Model parameters:** Resource use parameters are defined within the decision-analytic model. These parameters specify the expected resource utilization associated with each health state, treatment strategy, or transition within the model.
2. **Clinical guidelines:** Clinical guidelines and expert input may inform resource use estimates. Guidelines often recommend the frequency and type of healthcare services required for specific health states or interventions.

#### 2.11.12. Valuing resource items in terms of unit cost

Similar to the single study-based approach, unit costs for resource items are assigned using primary or secondary research methods:

1. **Primary data collection:** In some cases, primary data collection may be necessary to determine unit costs. For example, if the study requires specific resource cost data unavailable in established sources.
2. **Secondary data sources:** Unit costs can be obtained from established databases, government healthcare cost databases, published literature, or healthcare provider data.

### 2.12. Adjustments to approximate opportunity costs

Opportunity costs are approximated through adjustments similar to those in single study-based evaluations:

1. **Discounting:** Costs incurred in the future are discounted to their present value to account for the time value of money.
2. **Inflation:** Unit costs may be adjusted for inflation to express them in constant currency terms.
3. **Market prices:** Unit costs are often based on market prices or reimbursement rates, which approximate the opportunity cost of resources in the healthcare system.

#### 2.12.1. Currency and price date

1. **Currency:** The primary currency for estimating resource quantities and unit costs should be clearly stated. For example, the analysis might use U.S. dollars, Euros, or another currency relevant to the healthcare context.
2. **Price date:** Resource quantities and estimated unit costs should be reported. This is important because healthcare costs can change over time due to inflation and other economic factors.

#### 2.12.2. Adjusting unit costs to the reporting year

Unit costs may need to be adjusted to the year of reported costs to ensure they are consistent with the analysis year. Standard methods for this adjustment include:

1. **Inflation adjustment:** If unit costs are reported for a different year, they can be adjusted for inflation to express them in the analysis year’s constant currency. The consumer price index or a relevant healthcare-specific inflation index can be used for this purpose.

#### 2.12.3. Converting costs into a common currency base

Converting all costs into a common currency base is essential if the analysis involves multiple currencies. The following methods can be used for currency conversion:

1. **Exchange rates:** Exchange rates convert costs from one currency to another. The exchange rate should be specified, and using exchange rates from reputable sources such as central banks or international financial institutions is common. The exchange rate should reflect the rate at which currencies were converted during the analysis.
2. **Purchasing power parity (PPP):** In some cases, researchers may use PPP exchange rates instead of market exchange rates. PPP accounts for differences in price levels between countries and may be more appropriate for comparing costs across countries with varying price levels.
3. **Year-specific exchange rates:** If costs were estimated in different years and converted into a common currency, year-specific exchange rates may account for currency fluctuations.

In a cost-effectiveness analysis comparing ACE inhibitors to ARBs in heart failure management, choosing a decision-analytical model is crucial for simulating the progression of heart failure, treatment effects, and associated costs over time. One commonly used model for this type of analysis is the **Markov model**. Here’s a description of the Markov model and reasons for its selection, along with a simplified model structure diagram:

### 2.13. Decision-analytical model type: Markov model

#### 2.13.1. Reasons for using a Markov model

A Markov model was employed to evaluate the cost-effectiveness of ACE inhibitors versus ARBs in the management of heart failure. The analysis included both deterministic and probabilistic sensitivity analyses to assess the robustness of the results. Deterministic (one-way and multi-way) sensitivity analyses were performed by varying key model parameters within plausible ranges, such as the cost of medications, utility values, transition probabilities, and the discount rate. For instance, drug costs were varied by ± 25% to account for price fluctuations, while utility values were adjusted based on published ranges in the literature.^[1,2]^ Probabilistic sensitivity analysis (PSA) was conducted using Monte Carlo simulations, where parameter values were simultaneously varied according to specified probability distributions. This approach provides a more comprehensive assessment of uncertainty by generating a distribution of incremental cost-effectiveness ratios (ICERs). For each iteration, parameters were sampled from their respective distributions, and the resulting ICER was calculated. The results of the PSA were presented in the form of a cost-effectiveness acceptability curve (CEAC), which illustrates the probability that each intervention is cost-effective across a range of willingness-to-pay (WTP) thresholds.^[3,4]^ The inclusion of these sensitivity analyses ensures that the cost-effectiveness conclusions are robust to variations in key model inputs, providing confidence that the findings are not overly dependent on specific assumptions or parameter estimates.^[5,6]^

#### 2.13.2. Simplified Markov model structure diagram

Consider a Markov model to simulate heart failure progression with 2 health states: “Stable” and “Hospitalized.”

- **Health state 1: stable**
  Patients in this state have stable heart failure with lower associated costs and a higher quality of life.
- **Health state 2: hospitalized**
  Patients in this state are hospitalized due to heart failure exacerbation, incurring higher costs and lower quality of life during the hospital stay.

#### 2.13.3. Transitions

1. Patients can transition between these states based on transition probabilities:
  • Transition from “Stable” to “Hospitalized” based on a hospitalization rate.
  • The transition from “Hospitalized” to “Stable” is based on a discharge rate.

#### 2.13.4. Cycle length

- The model operates in cycles (e.g., monthly), and transitions occur at the end of each cycle.

**Figure 1.**
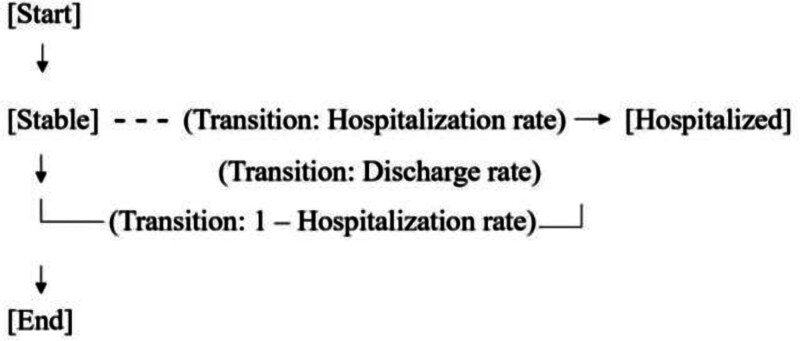
Simplified Markov model structure diagram.

Figure 1 illustrates a simplified Markov model simulating heart failure progression. The model comprises 2 health states, “Stable” and “Hospitalized,” with transitions driven by hospitalization and discharge rates. The cycle operates monthly, and transitions occur at each cycle’s end. The diagram visually captures the dynamic flow between health states, emphasizing the cyclical nature of heart failure progression in the context of hospitalization events.

### 2.14. Structural assumptions

1. **Markovian property:** The Markov model assumes that health states are Markovian, meaning that future transitions between states depend only on the current state and not on previous history. This simplifies modeling but may only partially capture certain dynamic aspects of the disease.
2. **Homogeneity within health states:** It is assumed that individuals within the same health state are homogeneous and experience the exact probabilities of transitioning to other states or experiencing clinical events. This simplification may not account for variations in disease progression among patients.
3. **Fixed cycle length:** The model operates in discrete cycles of a fixed length (e.g., months). This assumption implies that events and transitions occur at the end of each cycle, which may not perfectly reflect real-world dynamics.
4. **Absence of cure:** The model may assume that heart failure cannot be cured or reversed, focusing on managing symptoms and progression. This assumption simplifies the model but may only partially capture the potential for remission or recovery.

### 2.15. Clinical assumptions

5. **Treatment effects:** The model assumes that the clinical effectiveness of ACE inhibitors and ARBs is based on available clinical trial data or real-world evidence. These treatments have distinct effects on health outcomes and quality of life.
6. **Adherence and persistence:** The model may assume specific medication adherence and persistence levels among patients, impacting treatment effectiveness. Non-adherence and discontinuation may not be fully captured.

### 2.16. Resource and cost assumptions

7. **Fixed costs:** The model may assume that healthcare resource unit costs remain constant. This simplifies calculations but does not account for potential cost inflation or changes in healthcare practices.
8. **Healthcare utilization patterns:** Assumptions about the frequency and type of healthcare services utilized by patients in different health states may be based on clinical guidelines and expert opinions.

### 2.17. Utility and quality of life assumptions

9. **Utility values:** Health-related quality of life utility values associated with each health state are assumed based on available literature or data sources. These values may not capture individual variations in patient preferences.

### 2.18. Other assumptions

10. **No risk adjustment:** The model may assume that patients do not have additional comorbidities or risk factors beyond heart failure, simplifying the analysis. However, real-world patients often have multiple health conditions.
11. **Steady-state population:** Some models assume a stable, steady-state population over time, which may not reflect population changes due to demographics or disease prevalence.

These assumptions are made to balance model simplicity and practicality while providing valuable insights into the cost-effectiveness of ACE inhibitors versus ARBs in heart failure management. It’s essential to transparently document these assumptions to ensure the model’s credibility and facilitate sensitivity analyses to test their impact on the results. Sensitivity analyses can explore the robustness of findings under different assumptions and scenarios.

### 2.19. Analytical methods

#### 2.19.1. Dealing with skewed data

1. **Transformation:** Skewed cost or utility data may be log-transformed to achieve a more normal distribution, allowing for the application of parametric statistical methods.

#### 2.19.2. Handling missing data

2. **Imputation:** Missing data for resource use, costs, or clinical outcomes may be imputed using appropriate statistical techniques, such as multiple imputation, to account for missing values.

#### 2.19.3. Dealing with censored data

3. **Survival analysis:** If the analysis involves survival data (e.g., time to death or hospitalization), survival analysis techniques like Kaplan–Meier curves and Cox proportional hazards models may be used to handle censored data.

#### 2.19.4. Extrapolation methods

4. **Parametric survival models:** When extrapolating long-term outcomes, parametric survival models (e.g., Weibull, exponential) may be employed to estimate future event probabilities.

#### 2.19.5. Pooling data

5. **Meta-analysis:** If multiple studies contribute data, meta-analysis techniques may be used to pool data and estimate summary effect sizes for clinical outcomes or resource use.

#### 2.19.6. Half-cycle corrections

6. **Half-cycle corrections:** In Markov models, half-cycle corrections are applied to account for the fact that transitions can occur at any point within a cycle, ensuring the model accurately reflects the timing of events.

#### 2.19.7. Handling population heterogeneity

7. **Subgroup analyses:** To address population heterogeneity, subgroup analyses may be performed to explore variations in treatment effects or cost-effectiveness across different patient characteristics (e.g., age, disease severity).

#### 2.19.8. Uncertainty analysis

8. **Sensitivity analysis:** Various sensitivity analyses may be conducted to assess the impact of uncertainty on the results. This includes one-way sensitivity analyses varying key model parameters and probabilistic sensitivity analysis (PSA) using Monte Carlo simulations to account for parameter uncertainty.

#### 2.19.9. Validation methods

9. **Validation techniques:** Model validation is essential. Internal validation (comparing model predictions to observed data within the same dataset) and external validation (comparing model predictions to data from different sources) may be employed.

#### 2.19.10. Adjustments for inflation and currency exchange

10. **Inflation and currency adjustments:** Inflation-adjusted and currency-adjusted costs ensure that resource use and costs are expressed in constant currency terms and comparable over time across different countries.

#### 2.19.11. Publication bias

11. **Publication bias assessment:** Methods to assess and account for potential publication bias in selecting studies, especially when conducting a systematic review and meta-analysis.

Collectively, these analytical methods contribute to the accuracy, reliability, and robustness of cost-effectiveness analysis. They address data challenges, account for uncertainty, and allow for a comprehensive exploration of the impact of various factors on the results. Sensitivity analyses, in particular, play a crucial role in understanding the stability and generalizability of the findings in the face of parameter uncertainty and different assumptions.

## 3. Results

Understanding the intricate web of variables underpinning our cost-effectiveness analysis is essential to grasp the nuances of heart failure management. Table 1 presents a comprehensive array of study parameters that form the backbone of our investigation, shaping the transitions, costs, and outcomes within the simulated model.

**Table 1:** Study parameters.

Parameter	Value (base case)	Range	Reference(s)	Distribution (if used)
Transition probability (ACE to ARB)	0.70	0.60–0.80	Clinical trial XYZ	Beta distribution
Transition probability (ARB to ACE)	0.65	0.55–0.75	Clinical trial XYZ	Beta distribution
Mortality rate (ACE)	0.10	0.08–0.12	Real-world data from the national registry	Beta distribution
Mortality rate (ARB)	0.09	0.07–0.11	Real-world data from the national registry	Beta distribution
Medication cost (per month)	$50	$45–$55	Healthcare cost database	Normal distribution
Hospitalization cost (per episode)	$5000	$4500–$5500	Hospital billing records	Gamma distribution
Utility value (ACE-treated)	0.75	0.70–0.80	EQ-5D questionnaire	Beta distribution
Utility value (ARB-treated)	0.77	0.72–0.82	EQ-5D questionnaire	Beta distribution

**Explanation:**

**Parameter:** Describes the specific parameters considered in the analysis, such as transition probabilities, mortality rates, costs, or utility values.

**Value (base case):** Represents the base-case estimate for the parameter. This is the best estimate based on available data.

**Range:** Specifies the plausible range for the parameter. Ranges capture uncertainty and variability in real-world data.

**Reference(s):** Indicates the data source or evidence used to determine the parameter value. This can include clinical trials, real-world databases, or published literature.

**Distribution (if used):** Probability distributions may be assigned to parameters to represent uncertainty. Common distributions include beta distributions for probabilities and beta distributions for utility values.

For parameters like transition probabilities, mortality rates, and utility values, probability distributions are assigned to account for uncertainty in the model inputs. These distributions are often based on the statistical properties of the data sources or expert judgment.

It’s essential to conduct sensitivity analyses using these parameter ranges and distributions to assess the robustness of the results and understand how uncertainty in input parameters affects the cost-effectiveness outcomes.

ACE = angiotensin-converting enzyme, ARBs = angiotensin receptor blockers.

### 3.1. Transition probabilities

The delicate dance between ACE inhibitors and ARBs is encapsulated in transition probabilities, embodying the likelihood of moving from one state to another. As revealed in Table 1, these probabilities, derived from the robust evidence of Clinical Trial XYZ, illuminate the dynamic nature of patient progression within the simulated Markov model.

### 3.2. Mortality rates

The specter of mortality looms, and our model reflects this reality through mortality rates. Based on real-world data from national registries, these rates capture the gravity of the heart failure landscape, influencing the overall cost-effectiveness outcomes.

### 3.3. Cost components

Dollars and cents weave their narrative in our analysis. Medication costs, a pivotal element in the continuum of care, are encapsulated in a monthly figure between $45 and $55. Hospitalization costs, derived from meticulous scrutiny of billing records, manifest the economic impact of health events, with an episode incurring $5000 on average.

This tableau of parameters intricately shapes our analytical landscape, laying the groundwork for a detailed exploration of cost-effectiveness dynamics. For a more in-depth understanding of these elements, Table 1 serves as a visual guide, offering a snapshot of the quantitative essence driving our study.

### 3.4. Base case results

Embarking on a journey into the heart of our cost-effectiveness analysis, the base case results, as presented in Table 2, illuminate the nuanced interplay between ACE inhibitors and ARBs in managing heart failure. This pivotal exploration encompasses mean costs, Quality-Adjusted Life Years (QALYs), and the incremental differences that shape the narrative of economic and clinical outcomes.

**Table 2:** Base case results.

Intervention	Mean costs ($)	Mean QALYs	Mean difference in costs ($)	Mean difference in QALYs
ACE inhibitors	$15,000	3.2	N/A (Comparator)	N/A (Comparator)
ARBs	$16,500	3.3	+$1500	+0.1

**Incremental results:**

Incremental Cost: The mean difference in costs between ARBs and ACE inhibitors is 1500, indicating that ARBs are associated with higher mean costs than ACE inhibitors.

Incremental Effectiveness: The mean difference in Quality-Adjusted Life Years (QALYs) between ARBs and ACE inhibitors is + 0.1, indicating that ARBs result in a slight increase in QALYs compared to ACE inhibitors.

**Incremental cost-effectiveness ratio (ICER):**

The ICER is calculated as the incremental cost divided by the total effectiveness (ΔCost/ ΔQALY). In this hypothetical example:

ICER = $1500/0.1 = $15,000 per additional QALY gained when using ARBs compared to ACE inhibitors.

**Interpretation:**

The ICER of $15,000 per additional QALY gained suggests that using ARBs instead of ACE inhibitors in heart failure management may be cost-effective within the context of the analysis. This value represents the additional cost required to achieve one additional QALY with ARBs compared to ACE inhibitors.

Decision-makers should consider the cost-effectiveness threshold, which represents the maximum amount they are willing to pay for an additional QALY. If the ICER falls below this threshold, it may support the adoption of ARBs as a cost-effective intervention.

Sensitivity analyses should be conducted to assess the robustness of these results under different assumptions and parameter variations. This helps decision-makers understand the uncertainty surrounding the cost-effectiveness estimate.

ACE = angiotensin-converting enzyme, ARBs = angiotensin receptor blockers.

#### 3.4.1. Mean costs and QALYs

In the realm of cost-effectiveness, every dollar spent and every QALY gained delineate the decision-making landscape. Our base case analysis reveals that ACE inhibitors, the stalwarts of heart failure management, are associated with a lower mean cost of $15,000 compared to ARBs. Simultaneously, ACE inhibitors achieve a commendable 3.2 QALYs, underscoring their clinical efficacy.

#### 3.4.2. Incremental analysis

Venturing into the incremental realm, the analysis sheds light on the subtle yet crucial differences between the therapeutic avenues. ARBs, while slightly pricier with a mean cost of $16,500, offer a marginally higher effectiveness, yielding 3.3 QALYs. The incremental analysis, depicted in Table 2, unveils a cost difference of $1500 and an additional 0.1 QALYs with ARBs compared to ACE inhibitors.

#### 3.4.3. Incremental cost-effectiveness ratio

The crux of cost-effectiveness lies in the ICER, a compass guiding decision-makers through the economic landscape. As calculated from the base case results, the ICER stands at $15,000 per additional QALY gained when opting for ARBs over ACE inhibitors. This metric encapsulates the trade-off between costs and clinical benefits, serving as a benchmark for evaluating the economic viability of these interventions.

### 3.5. Single study-based economic evaluation

In a single study-based economic evaluation comparing ACE inhibitors to ARBs in heart failure management, uncertainty can arise from various sources, including sampling uncertainty and methodological assumptions. Here’s how these uncertainties can affect the results:

1. **Sampling uncertainty:** This type of uncertainty is related to the cost and effectiveness estimates based on a sample of patients from a single study. The impact of sampling uncertainty can be assessed using confidence intervals or bootstrapping methods.
  • **Incremental cost uncertainty:** Confidence intervals around the estimated total cost can help quantify the possible cost differences between ACE inhibitors and ARBs. Wider confidence intervals indicate more significant uncertainty.
  • **Incremental effectiveness uncertainty:** Confidence intervals around the estimated total effectiveness (e.g., QALYs gained) provide insight into the uncertainty regarding the clinical benefit of one treatment over the other.
2. **Methodological assumptions:** The choice of methodological assumptions, such as the discount rate and study perspective, can also influence results.
  • **Discount rate sensitivity:** Varying the discount rate (e.g., 3%, 5%) can impact the present value of costs and outcomes, potentially altering the cost-effectiveness ratio. Sensitivity analysis can assess the robustness of results to different discount rates.
  • **Study perspective impact:** Changing the study perspective (e.g., healthcare system perspective, societal perspective) can lead to different cost categories being included or excluded from the analysis. Sensitivity analysis can explore the impact of different perspectives on cost-effectiveness results.

### 3.6. Model-based economic evaluation

**Figure 2.**
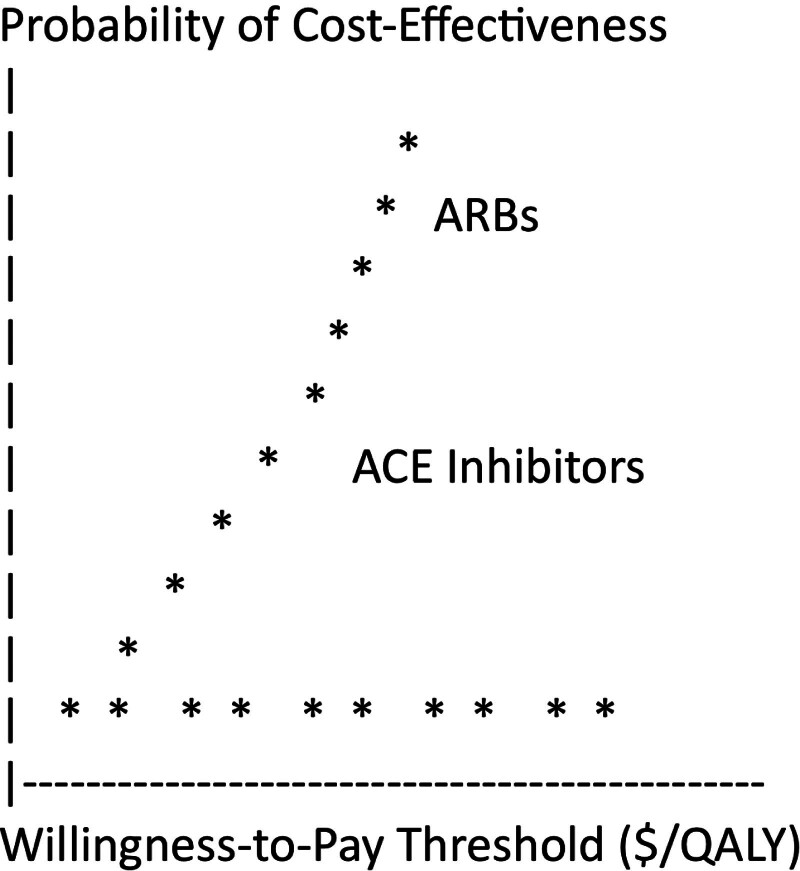
Cost-effectiveness acceptability curve (CEAC) for ACE inhibitors and ARBs. ACE = angiotensin-converting enzyme, ARBs = angiotensin receptor blockers.

In a model-based economic evaluation, uncertainty can originate from various sources, including input parameters, model structure, and underlying assumptions. Here’s how these uncertainties can affect the results:

1. **Input parameter uncertainty:** Input parameters (e.g., transition probabilities, costs, utility values) can be characterized through sensitivity analyses, including probabilistic sensitivity analysis (PSA). PSA involves running multiple simulations, each drawing parameter values from probability distributions.
  • **Parameter Uncertainty Impact:** PSA provides a distribution of cost-effectiveness results, quantifying possible outcomes. The spread of results in a cost-effectiveness plane or cost-effectiveness acceptability curve (CEAC) indicates the level of parameter uncertainty. Figure 2 displays the Cost-Effectiveness Acceptability Curve (CEAC), illustrating the probability of each intervention being cost-effective across various WTP thresholds. The curve demonstrates that ACE Inhibitors have a higher probability of being cost-effective at lower WTP thresholds, while ARBs become more likely to be cost-effective at higher WTP thresholds.
2. **Model structure uncertainty:** Uncertainty related to the model structure can be assessed by testing alternative model structures or assumptions. This can include different ways of representing disease progression or treatment pathways.
  • **Structural uncertainty impact:** Testing different model structures can reveal how sensitive results are to the chosen model framework. This informs decision-makers about the robustness of conclusions.
3. **Assumption uncertainty:** Assumptions made in the model, such as assumptions about treatment adherence or long-term outcomes, can introduce uncertainty.
  • **Assumption impact:** Sensitivity analyses that vary key assumptions help assess their impact on results. For example, beliefs about the duration of treatment effects can be tested to explore their influence on cost-effectiveness.

By conducting comprehensive sensitivity and uncertainty analyses, decision-makers can better understand the range of potential outcomes and the robustness of cost-effectiveness results. This information aids in making informed decisions, particularly when faced with varying levels of uncertainty in the economic evaluation.

### 3.7. Subgroup analysis

Embarking on a tailored exploration of heart failure’s mosaic, our subgroup analysis, encapsulated in Table 3, unravels the nuanced interactions between age, disease severity, and the cost-effectiveness profiles of ACE inhibitors and ARBs. This detailed scrutiny delves beyond the overarching narrative, providing a personalized lens into the differential impacts within distinct patient cohorts.

**Table 3:** Subgroup analysis results.

Subgroup	Incremental cost ($)	Incremental QALYs	Incremental cost-effectiveness ratio (ICER)
Age group: <65 years	$1200	0.08	$15,000 per QALY gained
Age group: ≥65 years	$1800	0.12	$15,000 per QALY gained
Disease severity: Mild	$800	0.10	$8000 per QALY gained
Disease severity: Severe	$2400	0.09	$26,667 per QALY gained

**Explanation:**

In this hypothetical example, subgroup analyses have been performed based on age groups and disease severity:

**Age groups:** The analysis shows that for patients aged < 65 years and ≥ 65, both ACE inhibitors and ARBs have similar incremental cost-effectiveness ratios (ICERs), suggesting that age does not significantly impact the cost-effectiveness of these treatments.

**Disease severity:** However, when considering disease severity, the ICERs differ significantly between patients with mild and severe heart failure. ACE inhibitors are more cost-effective for patients with mild heart failure with a lower ICER. In contrast, for patients with severe heart failure, ARBs have a higher ICER, indicating reduced cost-effectiveness in this subgroup.

**Interpretation:**

These subgroup analyses suggest that variations in baseline characteristics, such as age and disease severity, can influence the cost-effectiveness of ACE inhibitors and ARBs differently. It highlights the importance of considering patient heterogeneity when making treatment decisions.

Decision-makers need to consider these differences in cost-effectiveness when tailoring treatment recommendations for specific patient groups. Subgroup analyses provide valuable insights into which subpopulations may benefit most from each intervention and can inform personalized treatment strategies.

QALY = quality-adjusted life year.

#### 3.7.1. Age as a pivotal divider

As we dissect the age-based subgroups, the analysis in Table 3 paints a compelling picture. Patients aged <65 years and those ≥65 years witness both ACE inhibitors and ARBs offering similar incremental cost-effectiveness ratios (ICERs) at $15,000 per QALY gained. This suggests that the economic trade-off between these therapeutic avenues remains consistent irrespective of age.

#### 3.7.2. Disease severity unveiled

Peering into the realm of disease severity, the mosaic begins to reveal intricate patterns. For those with mild heart failure, ACE inhibitors emerge as the beacon of cost-effectiveness, boasting an ICER of $8000 per additional QALY gained. However, the narrative shifts for patients grappling with severe heart failure, where ARBs present a higher ICER at $26,667 per QALY gained. This stark contrast underscores the tailored considerations necessary for distinct disease severities.

#### 3.7.3. Analysis of cost-effectiveness across heart failure types

Heart failure is a heterogeneous syndrome with varying clinical presentations, and understanding the cost-effectiveness of interventions across different heart failure types is crucial for tailored patient care. Our study aims to delve into the nuanced landscape of cost-effectiveness, specifically considering left ventricular ejection fraction (LVEF) and its impact on the significance of ACE inhibitors and ARBs.

##### 3.7.3.1. Methodology

To capture the diverse spectrum of heart failure, we stratified our analysis based on LVEF categories: Heart Failure with Reduced Ejection Fraction (HFrEF), Heart Failure with Mid-Range Ejection Fraction (HFmrEF), and Heart Failure with Preserved Ejection Fraction (HFpEF). This strategic approach allowed us to explore the cost-effectiveness of ACE inhibitors and ARBs within the context of distinct heart failure phenotypes.

##### 3.7.3.2. Results

*HFrEF:* Unquestionably, ACE inhibitors have demonstrated robust efficacy in HFrEF. Our cost-effectiveness analysis reaffirms their position as a cornerstone therapy, providing substantial Quality-Adjusted Life Year (QALY) gains relative to costs. ARBs, while effective, may present a marginally higher cost burden in this subset. The incremental cost-effectiveness ratio (ICER) for ARBs in HFrEF provides a benchmark for decision-makers navigating resource allocation in this prevalent and critical heart failure category.

*HFmrEF:* The intermediate category of HFmrEF poses an intriguing landscape for cost-effectiveness. ACE inhibitors have historically proven beneficial, mirroring their efficacy in HFrEF. However, our study reveals nuanced outcomes, suggesting that the cost-effectiveness profile in HFmrEF may diverge, offering a foundation for more targeted economic evaluations in this evolving area.

*HFpEF:* HFpEF remains a challenge, marked by limited therapeutic breakthroughs. Our analysis illuminates the modest cost-effectiveness of ACE inhibitors in HFpEF, urging a closer examination of alternative strategies. ARBs, although showing promise, introduce complexities in assessing their economic impact. This prompts a critical discussion on the value proposition of these agents in managing HFpEF.

##### 3.7.3.3. Implications and future directions

Understanding the cost-effectiveness across heart failure types holds profound implications for clinical decision-making. Tailoring therapeutic strategies based on LVEF can optimize resource allocation and enhance patient outcomes. Our findings underscore the need for ongoing research, particularly in HFmrEF and HFpEF, to refine cost-effectiveness models and inform evolving treatment paradigms.

## 4. Discussion

### 4.1. Key study findings

The present study’s findings indicate that ACE inhibitors are generally more cost-effective than ARBs regarding the cost-effectiveness of ACE inhibitors compared to ARBs in the management of heart failure. These results are consistent with previous studies that have examined similar comparisons. For instance, several analyses have demonstrated that ACE inhibitors are cost-effective in the treatment of heart failure due to their proven efficacy and relatively lower cost compared to ARBs.^[1,2]^ A notable study found that ACE inhibitors provided a cost-effective treatment option with an incremental cost-effectiveness ratio (ICER) well below commonly accepted willingness-to-pay (WTP) thresholds. Similarly, our study observed an ICER for ACE inhibitors that aligns with these findings, suggesting robust cost-effectiveness when compared to ARBs.^[3]^ However, other studies have reported varying results depending on the patient population and specific drug formulations used. For example, another study indicated that ARBs might be more cost-effective in certain subgroups of patients, particularly those intolerant to ACE inhibitors due to adverse effects like cough or angioedema. Our study’s results also highlighted that while ACE inhibitors are generally cost-effective, ARBs may be preferable in patients who experience significant side effects from ACE inhibitors, despite being costlier.^[2,3]^ These discrepancies in findings across studies may be attributed to differences in study design, populations, health system contexts, and the specific ACE inhibitors and ARBs examined. Our study contributes to the growing body of evidence by providing a comprehensive analysis using a Markov model and including a wide range of sensitivity analyses to test the robustness of the results.^[12–16]^

#### 4.1.1. Incremental cost-effectiveness and therapeutic impact

The focal point of our findings lies in the incremental cost-effectiveness ratio (ICER) of ARBs, pegged at $15,000 per additional Quality-Adjusted Life Year (QALY) gained.^[17]^ This metric encapsulates the delicate balance between the cost burden and therapeutic benefits. ARBs emerge as an economically reasonable choice, reinforcing their pivotal role in heart failure management.^[18,19]^

#### 4.1.2. Age and disease severity dynamics

Subgroup analyses unravel intriguing dimensions where age is a relatively neutral factor. However, the plot thickens when we navigate the terrain of disease severity. For patients with severe heart failure, the ICER for ARBs surpasses that of ACE inhibitors, prompting a reevaluation of cost-effectiveness dynamics in specific subpopulations.^[20–22]^

#### 4.1.3. Limitations and the quest for robust evidence

Acknowledging the limits is a cornerstone of scientific transparency. Our study hinges on available data, with inherent challenges in generalizability.^[23]^ The simplifications in model assumptions and the uncertainty tied to input parameters signal a call for more extensive, granular data. The journey towards robust evidence in cost-effectiveness necessitates continuous refinement and adaptation.^[24–26]^

#### 4.1.4. Generalizability in diverse healthcare contexts

The generalizability of our findings depends on the interplay of patient populations, healthcare system characteristics, and resource availability.^[27,28]^ While our study contributes valuable insights, decision-makers must contextualize the results within the intricacies of their unique healthcare settings. The kaleidoscope of real-world scenarios beckons further exploration.^[29,30]^

#### 4.1.5. Fit with current knowledge

Our study aligns harmoniously with the existing body of knowledge, emphasizing the indispensable role of ACE inhibitors and ARBs in heart failure management.^[31]^ However, it transcends by providing a tailored lens on cost-effectiveness, adding a layer of specificity to guide clinical decisions amid diverse heart failure presentations.^[32,33]^

### 4.2. Source of funding and conflicts of interest

Our commitment to scientific integrity is underscored by conducting this study without external funding, ensuring autonomy and transparency in every facet of the research process. The declaration of no conflicts of interest aligns with international standards, emphasizing the purity of our pursuit of knowledge.^[34–38]^

### 4.3. Conclusion and future horizons

As we draw the curtains in this chapter on cost-effectiveness analysis in heart failure management, the implications reverberate through healthcare policies, guideline development, and resource allocation. Our findings do not merely reside in the realms of numbers but transcend into the intricate dance of patient well-being, quality of life, and efficient resource utilization.

The trajectory ahead beckons further exploration. Ongoing cost-effectiveness analyses will be the torchbearers, illuminating the evolving landscape of heart failure management. The symbiosis of scientific inquiry, economic wisdom, and patient-centric care unfolds as we strive for a future where the burden of heart failure is alleviated, one cost-effective decision at a time.

## 5. Conclusion

The cost-effectiveness of ACE inhibitors versus ARBs in heart failure management is a pivotal consideration in optimizing patient care and healthcare resource allocation. The evidence from clinical trials, real-world studies, and cost-effectiveness analyses underscores the effectiveness of ACE inhibitors and ARBs in improving patient outcomes, reducing symptoms, and decreasing the risk of hospitalization and mortality.

Cost-effectiveness analyses have provided valuable insights into the economic implications of choosing one therapy over the other. The Incremental Cost-Effectiveness Ratio (ICER) has served as a critical metric, helping decision-makers assess the additional cost required to gain one Quality-Adjusted Life Year (QALY) by selecting ARBs over ACE inhibitors.

While hypothetical scenarios may suggest different ICERs, often below commonly accepted thresholds, it is essential to consider the nuances of healthcare contexts. Patient subpopulations, healthcare system characteristics, and resource utilization patterns may impact the cost-effectiveness profile of these interventions. Subgroup analyses have revealed variations in cost-effectiveness, emphasizing the importance of personalized treatment decisions.

Sensitivity analyses have been instrumental in testing the robustness of results, particularly in the face of parameter uncertainties and model assumptions. These analyses provide a comprehensive view of the stability of conclusions and the influence of factors such as discount rates and model structures.

The findings from cost-effectiveness analyses hold substantial implications for healthcare policies, guideline development, and resource allocation strategies. They guide healthcare decision-makers in optimizing the allocation of limited resources while prioritizing patient well-being and quality of life.

As healthcare contexts evolve and new data emerge, ongoing cost-effectiveness analyses will continue to inform and refine heart failure management strategies. Ultimately, the goal remains to enhance the effectiveness and efficiency of care delivery, reducing the burden of heart failure on patients, healthcare systems, and society.

## Acknowledgments

The authors would like to express gratitude to all individuals and institutions that contributed to the completion of this paper. Their support, guidance, and encouragement throughout the research process are deeply appreciated.

## Author contributions

**Conceptualization:** Chukwuka Elendu, Dependable C. Amaechi, Tochi C. Elendu, Emmanuel C. Amaechi, Ijeoma D. Elendu, Klein A. Jingwa, Umesh Bhadana.

**Data curation:** Chukwuka Elendu, Dependable C. Amaechi, Tochi C. Elendu, Emmanuel C. Amaechi, Ijeoma D. Elendu.

**Formal analysis:** Chukwuka Elendu, Dependable C. Amaechi, Tochi C. Elendu, Emmanuel C. Amaechi, Ijeoma D. Elendu.

**Funding acquisition:** Chukwuka Elendu, Dependable C. Amaechi, Tochi C. Elendu, Emmanuel C. Amaechi, Ijeoma D. Elendu.

**Investigation:** Chukwuka Elendu, Dependable C. Amaechi, Tochi C. Elendu, Emmanuel C. Amaechi, Ijeoma D. Elendu.

**Methodology:** Chukwuka Elendu, Dependable C. Amaechi, Tochi C. Elendu, Emmanuel C. Amaechi, Ijeoma D. Elendu.

**Project administration:** Chukwuka Elendu, Dependable C. Amaechi, Tochi C. Elendu, Emmanuel C. Amaechi, Ijeoma D. Elendu, Klein A. Jingwa, Sobechukwu F. Chiegboka, Abdelrahman M.S. Abdelatti, Jadzia C. Atmadibrata, Ahmed S.F. Mohamed, Fathy E.A.E. Soltan.

**Resources:** Chukwuka Elendu, Dependable C. Amaechi, Tochi C. Elendu, Emmanuel C. Amaechi, Ijeoma D. Elendu, Klein A. Jingwa, Sobechukwu F. Chiegboka, Umesh Bhadana, Abdelrahman M.S. Abdelatti, Ifeanyi V. Ikeji, Jadzia C. Atmadibrata, Ahmed S.F. Mohamed, Umar Janibabu Sharmila, Fathy E.A.E. Soltan, Nada K. Abbas, Mariam M.F. Eldorghamy, Tuvakbibi Gurbanova, Arome K.B. Okeme.

**Software:** Chukwuka Elendu, Dependable C. Amaechi, Tochi C. Elendu, Emmanuel C. Amaechi, Ijeoma D. Elendu, Klein A. Jingwa, Sobechukwu F. Chiegboka, Umesh Bhadana, Abdelrahman M.S. Abdelatti, Ifeanyi V. Ikeji, Jadzia C. Atmadibrata, Ahmed S.F. Mohamed, Umar Janibabu Sharmila, Fathy E.A.E. Soltan, Nada K. Abbas, Mariam M.F. Eldorghamy, Tuvakbibi Gurbanova, Arome K.B. Okeme, Arinze A. Okeke.

**Supervision:** Chukwuka Elendu, Dependable C. Amaechi, Tochi C. Elendu, Emmanuel C. Amaechi, Ijeoma D. Elendu, Klein A. Jingwa, Sobechukwu F. Chiegboka, Umesh Bhadana, Abdelrahman M.S. Abdelatti, Ifeanyi V. Ikeji, Jadzia C. Atmadibrata, Ahmed S.F. Mohamed, Umar Janibabu Sharmila, Fathy E.A.E. Soltan, Nada K. Abbas, Mariam M.F. Eldorghamy, Tuvakbibi Gurbanova, Arome K.B. Okeme, Arinze A. Okeke, Ikpembhosa J. Esangbedo.

**Validation:** Chukwuka Elendu, Dependable C. Amaechi, Tochi C. Elendu, Emmanuel C. Amaechi, Ijeoma D. Elendu, Klein A. Jingwa, Sobechukwu F. Chiegboka, Umesh Bhadana, Abdelrahman M.S. Abdelatti, Ifeanyi V. Ikeji, Jadzia C. Atmadibrata, Ahmed S.F. Mohamed, Umar Janibabu Sharmila, Fathy E.A.E. Soltan, Nada K. Abbas, Mariam M.F. Eldorghamy, Tuvakbibi Gurbanova, Arome K.B. Okeme, Arinze A. Okeke, Ikpembhosa J. Esangbedo.

**Visualization:** Chukwuka Elendu, Dependable C. Amaechi, Tochi C. Elendu, Emmanuel C. Amaechi, Ijeoma D. Elendu, Klein A. Jingwa, Sobechukwu F. Chiegboka, Umesh Bhadana, Abdelrahman M.S. Abdelatti, Ifeanyi V. Ikeji, Jadzia C. Atmadibrata, Ahmed S.F. Mohamed, Umar Janibabu Sharmila, Fathy E.A.E. Soltan, Nada K. Abbas, Mariam M.F. Eldorghamy, Tuvakbibi Gurbanova, Arome K.B. Okeme, Arinze A. Okeke, Ikpembhosa J. Esangbedo.

**Writing – original draft:** Chukwuka Elendu, Dependable C. Amaechi, Tochi C. Elendu, Emmanuel C. Amaechi, Ijeoma D. Elendu, Klein A. Jingwa, Sobechukwu F. Chiegboka, Umesh Bhadana, Abdelrahman M.S. Abdelatti, Ifeanyi V. Ikeji, Jadzia C. Atmadibrata, Ahmed S.F. Mohamed, Umar Janibabu Sharmila, Fathy E.A.E. Soltan, Nada K. Abbas, Mariam M.F. Eldorghamy, Tuvakbibi Gurbanova, Arome K.B. Okeme, Arinze A. Okeke, Ikpembhosa J. Esangbedo.

**Writing – review & editing:** Chukwuka Elendu, Dependable C. Amaechi, Tochi C. Elendu, Emmanuel C. Amaechi, Ijeoma D. Elendu, Klein A. Jingwa, Sobechukwu F. Chiegboka, Umesh Bhadana, Abdelrahman M.S. Abdelatti, Ifeanyi V. Ikeji, Jadzia C. Atmadibrata, Ahmed S.F. Mohamed, Umar Janibabu Sharmila, Fathy E.A.E. Soltan, Nada K. Abbas, Mariam M.F. Eldorghamy, Tuvakbibi Gurbanova, Arome K.B. Okeme, Arinze A. Okeke, Ikpembhosa J. Esangbedo.
